# Discovery of a phylogenetically novel tropical marine Gammaproteobacteria elucidated from assembled genomes and the proposed transfer of the genus *Umboniibacter* from the family *Cellvibrionaceae* to *Umboniibacteraceae* fam. nov.

**DOI:** 10.3389/fmicb.2025.1437936

**Published:** 2025-03-28

**Authors:** Jia Yee Ho, Xiu Qi Koh, Deborah Yebon Kang, Adrian Low, Dalong Hu, Mindia A. S. Haryono, Rohan B. H. Williams, Rebecca J. Case, Yann Felix Boucher

**Affiliations:** ^1^Saw Swee Hock School of Public Health, National University of Singapore and National University Health System and National University Hospital System, Singapore, Singapore; ^2^Singapore Centre for Environmental Life Sciences Engineering (SCELSE), National University of Singapore, Singapore, Singapore; ^3^Centre for Translational Medicine, Department of Medicine, Yong Loo Lin School of Medicine, National University of Singapore, Singapore, Singapore; ^4^Singapore Centre for Environmental Life Science Engineering, Nanyang Technological University, Singapore, Singapore; ^5^School of Biological Sciences, Nanyang Technological University, Singapore, Singapore; ^6^Infectious Diseases Translational Research Program, Department of Microbiology and Immunology, Yong Loo Lin School of Medicine, National University of Singapore and National University Hospital System, Singapore, Singapore

**Keywords:** marine Gammaproteobacteria, a novel bacterial species, dissimilatory nitrate reduction, choline dehydrogenase, Singapore coast

## Abstract

Marine heterotrophic bacteria in coastal waters respond to the influx of carbon from natural and anthropogenic sources. We identified two nearly identical, (99.9% average nucleotide identity; 100% amino acid identity; same DNA G + C content of 52.3 mol%) high-quality (≥99% CheckM completeness and ≤ 1.3% contamination) draft metagenome-assembled genomes (MAGs; SJ0813 and SJ0972) from seawater microbiomes of a southern island of Singapore that is in a protected marine park. The MAGs were only assigned to the *Cellvibrionaceae* family according to Genome Taxonomy Database. Overall genome related indices to *Pseudomaricurvus alkylphenolicus* KU41G^T^ as the closest phylogenetic relative revealed no more than 70.45% average nucleotide identity (ANI_cutoff_ < 95%), below the 50% percentage of conserved proteins (POCP_cutoff_ = 43.54%) for genera cutoff and low digital DNA–DNA hybridization values (DDH = 20.6 and 20.8%). The major respiratory quinone is predicted to be ubiquinone-9 from the annotation of 3-demethylubiquinone-9 3-methyltransferase (*ubiG*, K00568) involved in the last step of the ubiquinone biosynthesis pathway (M00117), which differed from the ubiquinone-8 utilized by known members of *Cellvibrionaceae*. Both MAGs contained a complete pathway for dissimilatory nitrate reduction to ammonia, which increases bioavailability of nitrogen in seawater. An identical choline dehydrogenase found in both MAGs have a low amino-acid identity (≤64.47%) compared to existing GMC family oxidoreductases, expanding on the diversity of this family of enzymes. The MAGs meet nearly all the minimum requirements but lack a 16S rRNA gene of sufficient length required for the proposed novel genus and species under SeqCode. Nevertheless, phylogenetic trees based on core-genome and RpoB as an alternative phylogenetic marker are congruent with the taxon standing as a monophyletic clade to other taxa of the order Cellvibrionales. Taken together, the MAGs (SJ0813 and SJ0972) represent an uncultured, undescribed genus and species in which we tentatively propose the name *Candidatus* Pelagadaptatus aseana gen. nov., sp. nov. and strain SJ0813^TS^ (=BAABNI000000000.1^TS^) as type sequence. Phylogenetic inference from core-genome and RpoB phylogenetic trees placed *Umboniibacter marinipuniceus* KMM 3891^T^ outside *Cellvibrionaceae*. We, therefore, propose the transfer of the genus *Umboniibacter* from the family *Cellvibrionaceae* to a new family *Umboniibacteraceae* according to the International Code of Nomenclature of Prokaryotes.

## 1 Introduction

Coastal waters are dynamic environments subjected to nutrient influx from terrestrial and mixing from the open sea, and marine bacteria are proposed as indicators of environmental change (Pinhassi et al., 2022; Lønborg et al., 2021). The marine Gammaproteobacteria is a highly diverse class of bacteria consisting of distinct metabolic traits and growth strategies (Liu and Liu, 2020; Spring et al., 2015; Cho and Giovannoni, 2004). Over the years, the phylogeny of the Gammaproteobacteria class and particularly the Orders Alteromonadales, Cellvibrionales, Oceanospirillales, and Pseudomonadales have undergone several shifts and reclassifications based on core-proteins and-genes phylogenomic analyses (Liao et al., 2020; Williams et al., 2010). Within the Cellvibrionales order, are the families *Cellvibrionaceae, Halieaceae, Microbulbiferaceae, Porticoccaceae,* and *Spongiibacteraceae*. Most bacterial species of the *Cellvibrionaceae* characterized to date are found in the marine environment and are crucial players in the global carbon cycle as heterotrophic bacteria since they break down complex polysaccharides such as agarose, chitin, and cellulose, which constitute macroalgae and detritus fallen from coastal plants (Spring et al., 2015). The current *Cellvibrionaceae* family comprises 17 validly published genera of *Agaribacterium, Agarilytica, Cellvibrio, Eionea, Exilibacterium, Gilvimarinus, Halioxenophilus, Maricurvus, Marinimicrobium, Pseudomaricurvus, Pseudoteredinibacter, Saccharophagus, Sessilibacter, Simiduia, Teredinibacter, Thalassocella* and *Umboniibacter* with many representative species isolated from seawater or host-associated marine environments (Reimer et al., 2022; Spring et al., 2015; Parte et al., 2020). Within these, *Cellvibrionaceae* species *Cellvibrio japonicus*, *Gilvimarinus chinensis,* and *Marinimicrobium agarilyticum* have been shown to exhibit *r-*strategist traits that respond quicker to nutrient influx compared to *k*-strategists bacterial species in the *Halieaceae* and *Spongiibacteraceae* families, which form an oligotrophic marine gammaproteobacterial clade (Spring et al., 2015). The marine microbiota in Singapore is largely uncultured and surveys have mostly been conducted through 16S rRNA gene amplicon sequencing or microarray-based compositional analyses, albeit with limited phylogenetic resolution (Teramoto et al., 2013; Wijaya et al., 2023; Lau et al., 2013; Chenard et al., 2019; Deignan et al., 2023; Soh et al., 2024). It has been shown that the distribution of Gammaproteobacteria and Cellvibrionales differ around the coastal waters of Singapore and may be influenced by anthropogenic effects such as eutrophication and petroleum contamination (Teramoto et al., 2013; Chenard et al., 2019). Here, we describe two highly similar metagenome-assembled genomes (MAGs) belonging to a novel genus in the marine Gammaproteobacteria. The MAGs were from a spatial microbiome study of coastal seawater surrounding an island that is part of a marine conservation zone at South of Singapore. Furthermore, phylogenetic analysis of the order Cellvibronales suggests the creation of a novel family for the transfer of the validly recognized genus *Umboniibacte*r, which is currently classified under the family *Cellvibronaceae.*

## 2 Materials and methods

### 2.1 Sample collection and processing

Surface water samples were collected from two different locations through grab sampling (<1 m) along the coastal areas of St. John’s Island, Singapore (map grid reference: 1.216595 N, 103.850151 E) in August 2022, namely “Lagoon” (SJ0813; sample ID WLagoon_S196) and “Pier” (SJ0972; sample ID WPier_S191).” “Lagoon” serves as a frequented location where numerous visitors engage in swimming activities, and “Pier” serves as the designated area for the berthing of incoming and outgoing ferries. *In-situ* parameters of the water samples were measured using YSI ProQuatro Multiparameter Meter. Two liters of the collected water samples were filtered onto 0.22 μm sterile Cellulose Nitrate membrane filters (Sartorius, Göttingen, Germany) and extracted using the DNeasy® PowerWater® Kit (Qiagen, Hilden, Germany) following the manufacturer’s instructions.

### 2.2 Metagenomic sequencing, assembly, binning, and quality checks

Metagenomics sequencing of the extracted DNA samples was performed at the Singapore Centre for Environmental Life Sciences Engineering (SCELSE). The DNA libraries were constructed using TruSeq Nano DNA Library Kit and dual barcoded TruSeq DNA UD Indexes (Illumina, San Diego, USA) before 150 bp paired-end sequencing reads were generated using the Illumina HiSeqX platform. An average of 18.3 Mbp raw sequencing reads per sample were obtained (350 Mbp for 19 samples). Raw reads were filtered using the Kneaddata pipeline v0.7.7^1^ where human contamination and low-quality reads were filtered before constructing the initial assembly via metaSPAdes v3.13.0 with default parameters (Nurk et al., 2017). Additionally, after initial raw reads quality step, MetaPhlAn 4 (database: vJan21_CHOCOPhlAnSGB_202103) was used for profiling the composition of microbial communities to draft taxonomic assignments and estimation of relative abundance (Blanco-Míguez et al., 2023). Subsequently, the binning, bin refinement, bin reassembly, and initial bin taxonomy classification were performed using the metaWRAP pipeline v1.3 with the quality threshold for the bins set at >50% completeness and < 10% contamination rate (Uritskiy et al., 2018). The taxonomy of the final bins was further assessed using Genome Taxonomy Database toolkit (GTDB-Tk) v2.1.1 using the Genome Taxonomy Database release 214 (Chaumeil et al., 2022). MAGs were accessed for completeness and contamination using CheckM v1.2.2 (Parks et al., 2015). To estimate the abundance of our novel *Cellvibrionaceae* MAGs in the metagenomic reads, trimmed raw reads of each sample were mapped back to the *Candidatus* Pelagadaptatus aseana MAGs using Bowtie2 v2.5.3 with default settings (Langmead and Salzberg, 2012). The relative abundance of the MAGs was calculated by dividing the number of mapped reads by the total number of reads for the sample.

### 2.3 Genome annotation and phylogenetic analysis

For all software, default parameters were selected unless otherwise specified. Prokka v1.14.5 was used to predict protein-coding sequences (CDS) and RNA, and the resulting protein and nucleotide sequences were applied to downstream applications (Seemann, 2014). The predicted protein sequences were annotated using Kyoto Encyclopedia of Genes and Genomes (KEGG) database release 108.1 and BlastKOALA (accessed 24 Jan 2024) (Kanehisa et al., 2016; Kanehisa et al., 2023). Panaroo v1.3.2 was used to find and align (MAFFT) the core genes among the RefSeq genomes with changes to the following parameters (−-clean-mode strict, −f 0.5, −c 0.85, −-len_dif_percent 0.8, and --core_threshold 1.0, −-aligner mafft) (Tonkin-Hill et al., 2020; Katoh and Standley, 2013; Katoh et al., 2002). Genomes selected for comparison are those of the earliest published species for each genus. The aligned concatenated core genes was used to build a maximum-likelihood tree with RAxML-NG using the GTR + G model, 12,345 as seed with bootstrapping performed using Transfer Bootstrap Expectation (TBE) method (Kozlov et al., 2019). RpoB protein sequences were aligned using MUSCLE in MEGA-X followed by RAxML-NG using the LG + G model, 12,345 as seed with bootstrapping performed using TBE method (Kozlov et al., 2019; Edgar, 2004; Kumar et al., 2018). Average nucleotide identity (ANI) using BLAST algorithm was performed using JSpeciesWS (Richter et al., 2016). Average amino acid identity (AAI) was performed using CompareM v0.1.2^2^. The Percentage of Conserved Proteins (POCP) was analysed using POCP calculator^3^ (Qin et al., 2014). DNA–DNA hybridization values were calculated using formula 2 (identities/high scoring segment pair length) in the Genome-to-Genome Distance Calculator v3.0^4^ (Meier-Kolthoff et al., 2021). Taxonomic nomenclature and etymology were based on suggestions using Protologger v1.0 on Galaxy (accessed 24 Jan 2024) and published guidelines (Hitch et al., 2021; Trüper, 1999; Galaxy, 2022). Protein BLAST (blastp) of the RpoB sequence and choline dehydrogenase was performed against NCBI metagenomic proteins (10,979,702 sequences) and non-redundant protein databases (717,423,220 sequences) (both accessed 25 March 2024) (Altschul et al., 1997; Altschul et al., 2005). The accession numbers for all the genomes and RpoB sequences are provided in Supplementary Table S1. Proksee was used to generate the plot for genome comparison (Grant et al., 2023).

### 2.4 SSU rRNA and *rpoB* identification from binned and unbinned reads

Infernal v1.1.5 was used (cmsearch --tblout) to identify SSU rRNA genes from assembled contigs (binned or unbinned) (Nawrocki and Eddy, 2013). Sequences with length > = 1,125 bp (SeqCode 16S rRNA gene requirement >75% complete) were shortlisted. The start (seq from) and end (seq to) position of 16S rRNA sequence detected by Infernal was used to extract the 16S rRNA sequence from the MAGs with samtools faidx (samtools v1.6) (Danecek et al., 2021). Extracted sequences were then aligned to 16S_ribosomal_RNA database downloaded from NCBI (modified 27-08-2024, downloaded 06-11-2024) using BlastN v2.15.0+ (−outfmt 7), and taxonomic information (Organism and Family) were manually added according to the NCBI accession ID (Sayers et al., 2021). To find the *rpoB* sequence of SJ0972 (WPier_S191), we used the *rpoB* gene sequence of SJ0813 as a template to search the unbinned reads using BlastN v2.16.0 (−outfmt 6) (Sayers et al., 2021).

## 3 Results

### 3.1 Phylogenomic analysis of phylogenetically novel MAGs

**Figure 1:**
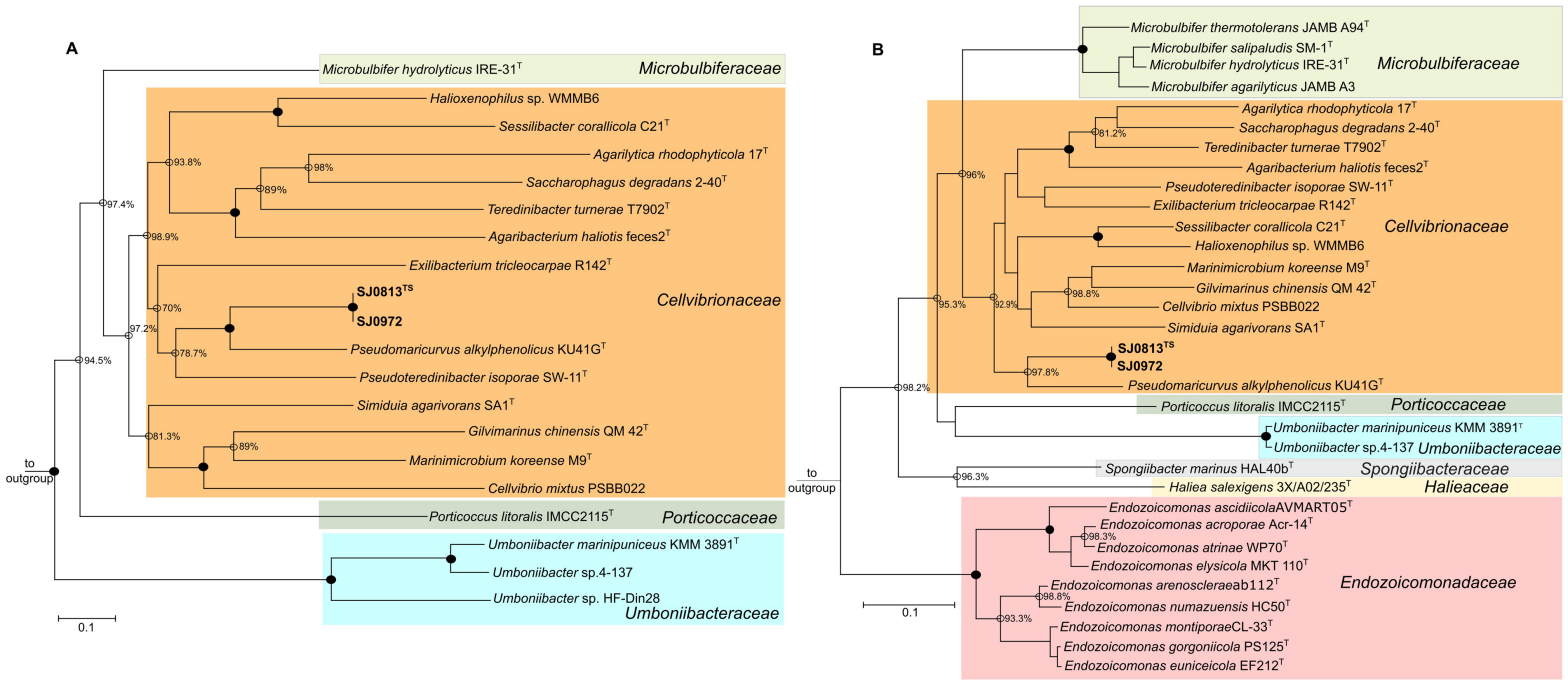
Maximum-likelihood trees of selected Gammaproteobacteria. **(A)** Core-genome tree of on 20 core genes belonging to 22 genomes of the type species for each genus including two addition reference genomes of *Umboniibacteraceae* of the Cellvibrionales order. The phylogenome tree is rooted to *Haliea salexigens* and *Microbulbifer hydrolyticus* as outgroup. **(B)** A phylogenetic tree of RpoB sequences to resolve *Umboniibacter* phylogeny. RpoB protein sequence of SJ0972 is partial (1,106 aa), while *Umboniibacter* sp.4–137 lacked the RpoB protein sequence. The RpoB tree is rooted to *Escherichia coli* ATCC 11775^T^ (RefSeq WP_000263098.1) as outgroup and is not shown. For both trees, the scale bar indicates 0.1 nucleotide or amino acid substitution per site. Family lineages are color coded. Filled nodes denote 100% bootstrap (transfer bootstrap expectation) values and open nodes are bootstrap values of at least 70%. NCBI/DDBJ RefSeq or GenBank accession numbers are provided in Supplementary Table S1.

Two high-quality (≥99% completeness and ≤ 1.3% contamination) draft MAGs of 3.46 Mbp (SJ0813) and 3.45 Mbp (SJ0972) assembled with read coverage (≥61×) were sequenced from seawater collected from two locations around St. John’s Island, Singapore. The MAGs are highly similar to each other with 99.99% ANI and AAI (Table 1 and Supplementary Figure S1). Phylogenetic analysis using the GTDB classifier only assigned the MAGs to the family *Cellvibrionaceae* with multiple sequence alignments showing ≤83.22% protein identity to the closest genome and relative evolutionary divergence (≤0.883) (Table 1). A maximum-likelihood tree was constructed using 20 single copy core genes (100%) of type strains and/or representative sequence genomes (RefSeq) (*n* = 16) in *Cellvibrionaceae* as well as one type species from the remaining four families within the order Cellvibronales: *Halieaceae, Microbulbiferaceae, Porticoccaceae,* and *Spongiibacteraceae* (*n* = 4). The phylogeny supports that the MAGs belong to the *Cellvibrionaceae* lineage but are yet distinct from their closest relative, *Pseudomaricurvus alkylphenolicus* KU41G^T^ (Figure 1A). Moreover, each MAG (SJ0813 and SJ0972) contained an identical 143 bp segment belonging to the start of the 16S rRNA gene (V1 regions) with closest BLAST hit (95.1% identity) to *Cellvibrio japonicus* Ueda107 of the family *Cellvibrionaceae* (Supplementary Table S2). Comparative alignment of the short SSU rRNA genes to *C. japonicus* Ueda107, *Cellvibrio mixtus* ACM 2601^T^, and *P. alkylphenolicus* KU41G revealed identities of 93.9, 90.5 and 90.5%, which are less than the threshold (94.5%) for genus cutoff for full-length SSU rRNA gene, as previously proposed (Yarza et al., 2014). Further attempts to find a SSU rRNA gene to fulfill the requirement of SeqCode (~1,250 bp or > 75% of complete SSU rRNA gene) in the binned and unbinned contigs using the Infernal pipeline, yielded 21 sequences that do not belong to any bacterial species of *Cellvibrionaceae* (Supplementary Table S3). As an alternative marker, we utilized RpoB to build a maximum-likelihood tree to show that the MAGs are indeed their own monophyletic clade (Figure 1B). This relationship is congruent to the RpoB maximum-likelihood tree as shown in Figure 1A. It should be noted that the SJ0972 *rpoB* gene was only partial (3,318 bp) albeit with 100% similarity to the *rpoB* of SJ0813 (4,085 bp), and both (1,361 aa for SJ0813^TS^; 1,106 aa for SJ0972) were included in the RpoB phylogenetic tree. The POCP comparison to *P. alkykphenolicus* is no more than 43.54%, ANI comparison to *P. alkylphenolicus* KU41G^T^ shows no more than 70.45% identity and 39.80% coverage and low digital DDH of 20.6 and 20.8% (Table 1).

**Table 1:** Statistics and quality of metagenome-assembled genomes compared to the closest known bacterial species.

Parameter	SJ0813	SJ0972	*Pseudomaricurvus alkylphenolicus* KU41G^T^
Genome size (bp)	3,461,300	3,454,785	8,879,859
No. of Contigs	25	21	71
G + C content (mol%)	52.3	52.3	53.5
Completeness (%)	99.53	98.98	96.88
Contamination (%)	1.293	0.862	2.06
Contig N50 value (bp)	262,903	429,412	392,482
Protein coding genes (CDS)	3,117	3,110	7,212
tRNA gene (no. of unique)	29 (16)	30 (17)	46 (20)
rRNA gene (5S, 16S, 23S)	0, 1 (143bp), 0	1, 1 (143 bp), 0	4, 4 (1 complete, 3 partial), 5 (partial)
Genome coverage	61.8×	155.2×	67.6×
ANI (%), alignment (%) to *SJ0813*	–	99.99, 98.89	70.45, 39.80
ANI (%), alignment (%) to *SJ0972*	99.97, 99.02	–	70.25, 39.66
Mean AAI (%), orthologous fraction (%) to *Pseudomaricurvus alkylphenolicus* KU41G^T^	66.68, 67.87	66.60, 67.79	–
POCP value (%) to *Pseudomaricurvus alkylphenolicus* KU41G^T^	43.54	43.35	–
Digital DDH (%) to *Pseudomaricurvus alkylphenolicus* KU41G^T^	20.8	20.6	–
GTDB multiple sequence alignment (%)	89.04	83.22	–
GTDB relative evolutionary divergence	0.883	0.883	–
DDBJ/GenBank Accession No.	BAABNI000000000.1	BAABNJ010000001	GCF_011683955.1
SeqCode ID	34343	–	–

### 3.2 Description of characteristic respiratory, flagella and metabolic proteins

**Figure 2:**
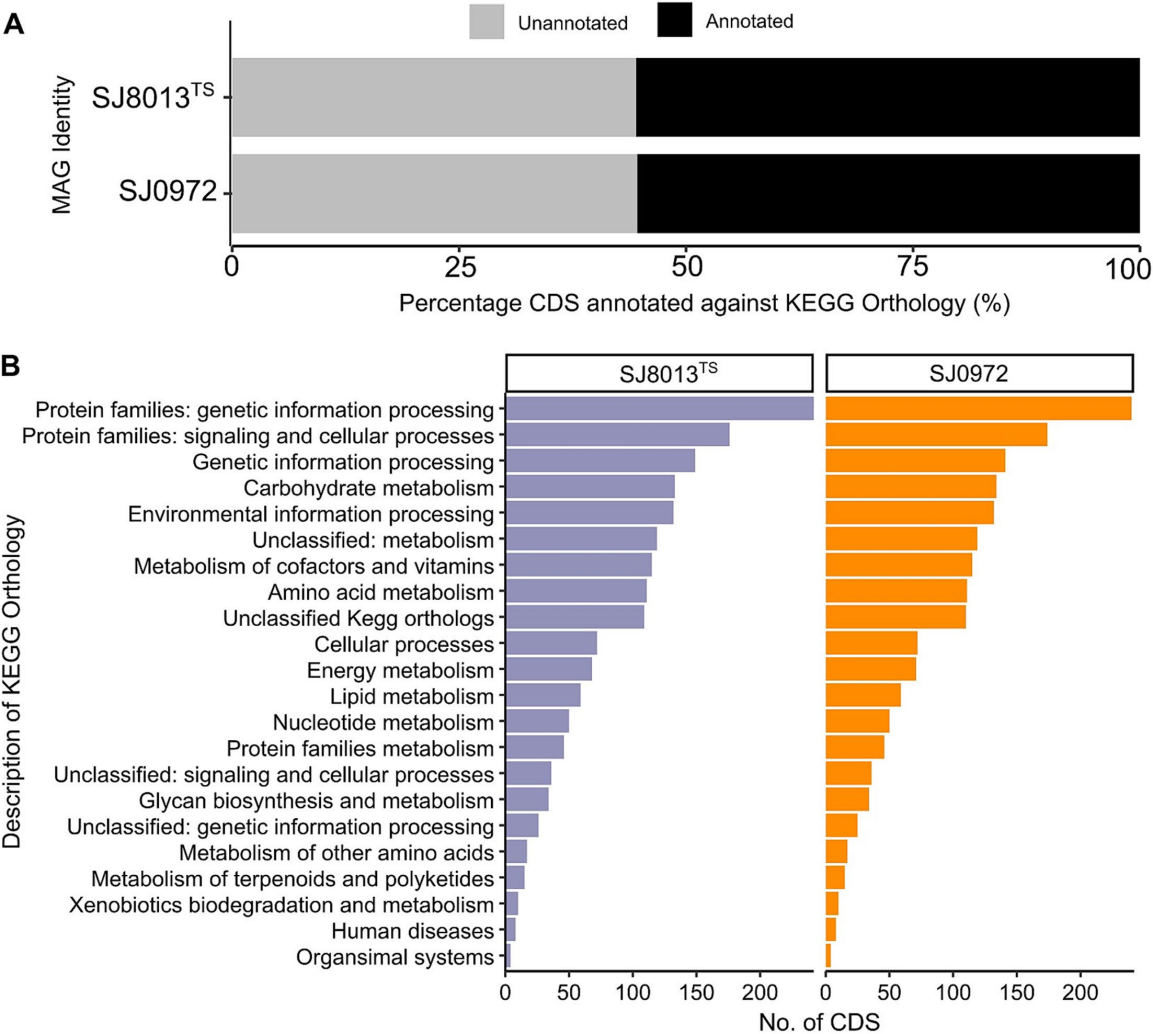
Annotation of coding sequence (CDS) using KEGG Orthology (KO) for both the MAGs (SJ0813 and SJ0972). **(A)** Percentage of CDS annotated and unannotated by KO. **(B)** Number of CDS assigned to major subsystems of KEGG modules, including proteins related to genetic information processing and signaling and cellular processes, genetic information processing, carbohydrate metabolism, environmental information processing, metabolism of cofactors and vitamins, amino acid metabolism, cellular processes, energy metabolism, lipid metabolism, nucleotide metabolism, protein families metabolism, glycan biosynthesis and metabolism, metabolism of other amino acids, metabolism of terpenoids and polyketides, xenobiotics biodegradation and metabolism, human diseases and organismal systems as well as some unclassified KO.

We annotated the CDS of the novel MAGs against the KEGG orthology (KO). Similar proportion of CDS of both MAGs matched a KO protein (Figure 2A) and the major subsystems are proteins related to genetic information processing and signaling and cellular processes (Figure 2B). The complete KEGG modules and KOs of both MAGs are listed in Supplementary Table S4. The presence of the citrate cycle pathway and the dissimilatory nitrate reduction to ammonia may be indicative of both aerobic and anerobic respiratory mechanisms. Other features include *ubiG* encoding for a 3-demethylubiquinone-9 3-methyltransferase in the ubiquinone biosynthesis pathway (M00117) may express ubiquinone-9 (Q9) as the predominant lipoquinone. Proteins that encode for chemotaxis (KEGG pathway map02030, 15 proteins) and flagella assembly (KEGG proteins map02040; 38 proteins) suggest motility of planktonic cells. Additionally, both our MAGs had unique protein sequence of choline dehydrogenase, which is identical to each other but with only 64.47% amino acid identity to a Glucose-Methanol-Choline (GMC) family oxidoreductase belonging to an uncultured *Colwelliaceae* sp. BS250 (GenBank MEW6996986.1). When matched against choline dehydrogenase sequence of *P. alkylphenolicus* KU41G^T^ (GenBank WP_166985401.1), the identity was only 43.28%. Moreover, both genomes have complete carbohydrate metabolic pathways, glycolysis (M0002), pyruvate oxidation (M00307), pentose phosphate pathway (M00007), and PRPP biosynthesis (M00004).

### 3.3 Local and global distribution of the novel MAGs

To ascertain the global distribution of the novel MAGs, we ran protein BLAST of the RpoB sequence (SJ0813^TS^) against the NCBI metagenomic proteins (env_nr) and non-redundant protein sequences (nr) to leverage global sequence archive of the International Nucleotide Sequence Database Collaboration. The RpoB sequence matched 80.66% identity (99% query coverage) to a RpoB of a deep marine sediments metagenome (GenBank accession no. KKN45857.1) and 90.15% identity (99% query coverage) to a RpoB (GenBank accession no. MAZ90271.1) of a MAG (TOBG_SAT-84) sequenced from marine surface water, respectively (Spang et al., 2015; Tully et al., 2018). However, the MAG (TOBG_SAT-84) was too low in quality (draft genome of 1.17 Mbp, 64.32% completeness, 1.15% contamination) to make a further comparisons (Tully et al., 2018). From the BLAST Atlas comparison, the *rpoB* gene of SJ0813 showed no similarity to that of *P. alkylphenolicus* (Supplementary Figure S1).

### 3.4 Diversity of seawater microbiome

**Figure 3:**
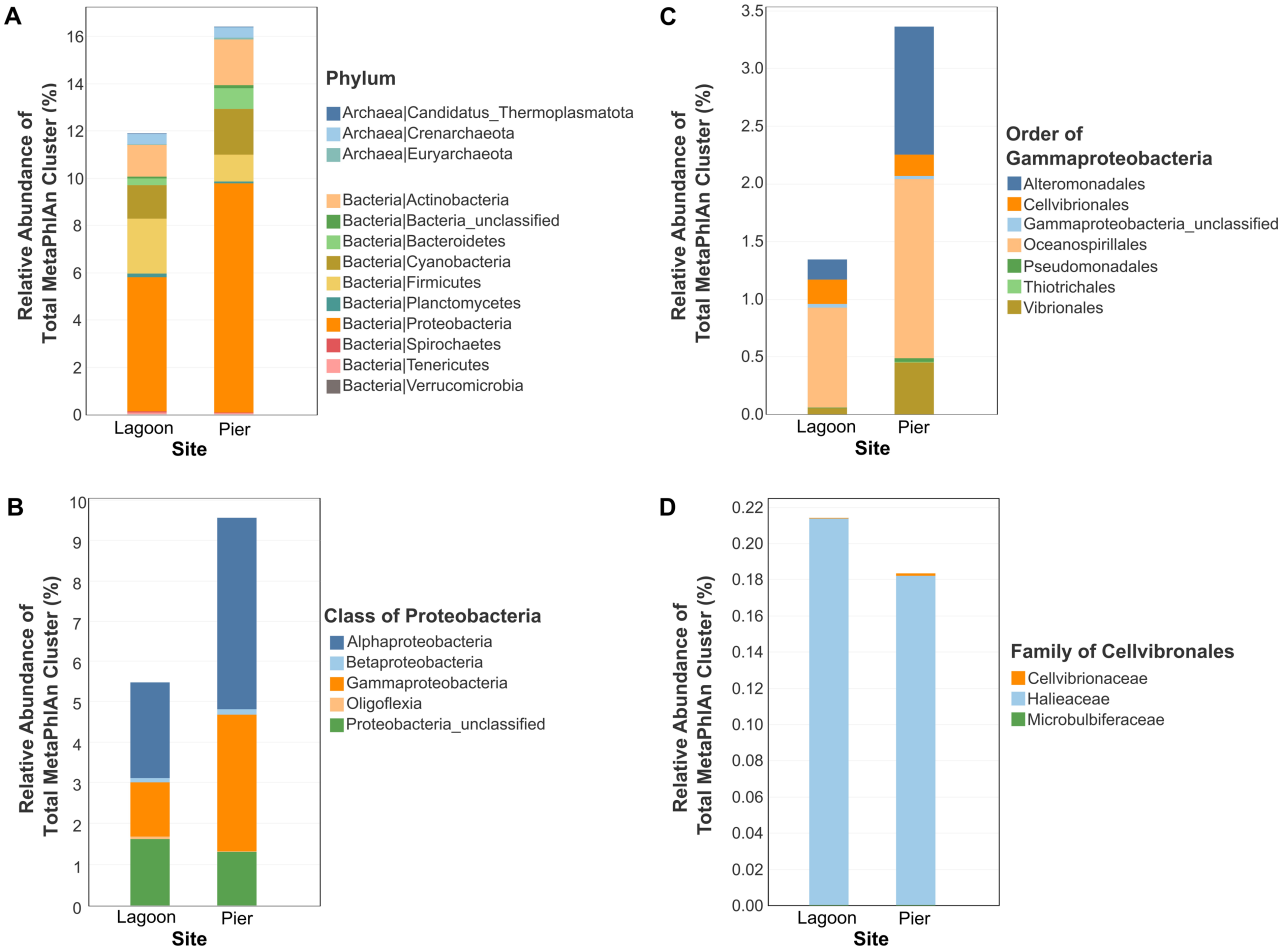
The relative abundance of a MetaPhlAn cluster to total MetaPhlAn cluster for lagoon and pier samples at different taxonomic levels analyzed using MetaPhlAn 4. **(A)** An overall microbial community at the phylum level for archaea and bacteria. **(B)** Diversity within the Proteobacteria at the class level. **(C)** Diversity within Gammaproteobacteria at the order level. **(D)** Diversity within Cellvibronales at the family level. Barplots depicting taxa with low relative abundance is diminutive to discern.

The *in-situ* parameters of seawater reflect in the lagoon and pier are similar at the point of sampling for Temperature (°C), dissolved oxygen (mg/L), conductivity (mS/cm), total dissolved solid (TDS) (mg/L), salinity (ppt) and pH (Table 2). The water temperature is similar to those recorded for St. John’s Island in the Marine Environment Sensing Network’s Ombak database, ranging from 24.2°C to 32.0°C (December 2022 to September 2024 except August 2022 where data was not available). We characterized the microbiomes of seawater of a lagoon (SJ0813) and a pier (SJ0972) and obtained a taxonomic profile using MetaPhlAn 4. A large proportion of the sequencing reads were not assigned to either bacteria or archaea for both the samples, lagoon (88.11%, relative abundance of MetaPhlAn cluster to total MetaPhlAn cluster) and pier (83.58%) sites. At the phylum level, Pseudomonadota (previously known as Proteobacteria) have the highest relative abundance (5.68% at the lagoon and 9.69% at the pier), followed by Firmicutes at the lagoon (2.34%) and Cyanobacteria at the pier (1.94%) (Figure 3A). Within Proteobacteria, Alphaproteobacteria (lagoon = 2.36% and pier = 4.72%) and Gammaproteobacteria (lagoon = 1.34% and pier = 3.36%) are the predominant class in both microbiomes (Figure 3B). Within Gammaproteobacteria, the most abundant Order is Oceanospirillales (lagoon = 0.87% and pier = 1.55%) followed by Cellvibrionales (0.21%) at the lagoon or Alteromonadales (1.11%) at the pier (Figure 3C). Within Cellvibrionales, the family *Halieaceae* was the most abundant with *Cellvibrionaceae* ≤ 0.001% as a minor population (Figure 3D). The relative abundance of our novel *Cellvibrionaceae* MAGs was estimated by mapping the raw reads onto *Candidatus* Pelagadaptatus aseana. In the lagoon and pier, the relative abundance of *Candidatus* Pelagadaptus aseana MAGs to total metagenomic reads was 2.72 and 6.25%, respectively.

**Table 2:** *In situ* physiochemical parameter measurements at the sampling locations.

Site	Temperature (°C)	Dissolved oxygen (mg/L)	Conductivity (mS/cm)	Total dissolved solid (TDS) (mg/L)	Salinity (ppt)	pH
Pier	29.4	4.92	51.7	31005.00	30.93	8.45
Lagoon	29.4	4.98	51.7	31005.00	30.92	8.59

### 3.5 The proposed transfer of *Umboniibacter* from *Cellvibrionaceae* to a new family *Umboniibacteraceae*

Interestingly, the maximum-likelihood phylogenetic tree based on core-genome showed that the formerly described *Umboniibacter marinipuniceus* KMM 3891^T^ and undescribed *Umboniibacter* spp. strains 4–137 and HF-Din28 fell outside of *Cellvibrionaceae* clade and are more closely related and yet distinct from *Porticoccus litoralis* IMCC 2115^T^ (*Porticoccaceae*), *Haliea salexigens* 3X/A02/235^T^ (*Halieaceae*), and *Spongiibacter marinus* HAL40b^T^ (*Spongiibacteraceae*) (Figure 1A). We constructed the maximum-likelihood tree using RpoB and included nine *Endozoicomonas* spp. and four *Microbulbifer* species, alongside the taxa from the core-genome tree. Like the core-genome phylogenetic tree, *U. marinipuniceus* KMM 3891^T^ and *Umboniibacter* sp. strain 4–137 formed a distinct clade from outside of the families *Cellvibrionaceae* and *Porticoccaceae, Halieaceae*, *Spongiibacteraceae* and *Endozoicomonadaceae* (Figure 1B). The AAI (57.2, 46.31% orthologous fraction) and ANI 65.46, 20.24% coverage of *P. litoralis* IMCC 2115^T^ and *U. marinipuniceus* KMM 3891^T^ support their delineation belonging to different genera and species, respectively. Based on the phylogeny of *U. marinipuniceus* KMM 3891^T^ and its close relatives, *Umboniibacter* spp. strains 4–137 and HF-Din28, which form a monophyletic clade and are distinct from other members in the Order Cellvibrionales. We proposed transfer of *Umboniibacter* from the family *Cellvibrionaceae* to a newly proposed family, *Umboniibacteraceae* based on the International Code of Nomenclature of Prokaryotes (ICNP).

## 4 Discussion

The tropical marine microbiota diversity of South-East Asia is still largely uncharacterized and previous studies of Singapore waters using the 16S rRNA gene lacked phylogenetic resolution (Teramoto et al., 2013; Wijaya et al., 2023; Lau et al., 2013; Chenard et al., 2019; Deignan et al., 2023; Soh et al., 2024). In this study, we used metagenomics to describe the genome and phylogeny of an uncultured marine bacteria, which was found in two separate locations surrounding an island at South of Singapore, the St. John’s Island. This island is part of a marine protection zone that has a rich diversity of corals, which serve as spawning sites and nursery grounds for marine life and thus are economically important for the fishery industry (NG and CHOU, 2017).

The MAGs are phylogenetically novel based on the different phylogenomic measurements, genome assembly quality criteria, and predicted biochemical pathways. POCP and ANI comparison of our MAGs to *P. alkylphenolicus* was well below the 50% genus boundary (Qin et al., 2014) and 95% bacterial species ANI threshold (Goris et al., 2007; Parks et al., 2020). A majority of species within Cellvibrionales order have ubiquinone-8 (Q8) as the major respiratory lipoquinone (Spring et al., 2015) in contrast with our novel MAGs, which may express ubiquinone-9 (Q9) as the predominant lipoquinone. This lipoquinone is synthesized by 3-demethylubiquinone-9 3-methyltransferase (*ubiG*, K00568) in the ubiquinone biosynthesis pathway (M00117; 9 proteins), which includes the three ubiquinone flavin-dependent monooxygenases, UbiH, UbiI, and UbiF (Kazemzadeh et al., 2023; Kanehisa et al., 2023). Notably, *Pseudoteredinibacter isoporae*, which is basal to the clade grouping *P. alkylphenolicus* and our novel MAG (Figure 1) also predominantly express Q9 lipoquinone (Spring et al., 2015; Chen et al., 2011). It should be noted that the respiratory quinones are inferred from *in silico* annotations and would still require biochemical analysis to validate the phenotype (Vieira et al., 2021). The carbohydrate and nitrogen metabolism based on complete KEGG modules suggest a chemoheterotrophic lifestyle capable of utilizing glucose through the citrate cycle but may be capable of anaerobic respiration through dissimilatory nitrate reduction (Einsle et al., 1999). The gene, nitrate reductase (NapAB) and nitrite reductase (NirBD) which is essential for the reduction are present in both our MAGs and *P. alkylphenolicus*. Incidentally, while *P. alkylphenolicus* KU41G^T^ also possess the genes for NapAB and NirBD, it did not exhibit nitrate reduction to nitrite when biochemically tested (Seo et al., 2015). Hence, the biochemical pathways described here remain as predictions until there is empirical evidence. Dissimilatory nitrate reduction is a major pathway in coastal zones for the preservation of nitrogen in the form of ammonium that is source of nitrogen for sea plants and is an advantageous trait during low oxygen levels as a result of eutrophication, which are common around coastal waters and off-shore fisheries (Gillis et al., 2017). The discovery of a choline dehydrogenase with low sequence identity to existing GMC oxidoreductase of an uncultured *Colwelliaceae* marine bacterium expands the sequence diversity of the GMC superfamily, which is found in all kingdoms of life (Ivanova et al., 2004; Aleksenko et al., 2020). Choline dehydrogenase catalyzes the oxidation of choline to glycine-betaine, which has several biochemical functions, including being an osmolyte for marine bacterial adaptation to salinity in seawater and a precursor in the methionine cycle (Mausz et al., 2022; Gadda and McAllister-Wilkins, 2003; Yancey, 2005). Taken together, we tentatively proposed a novel bacterial genus and species called *Candidatus* Pelagadaptatus aseana gen. nov., sp. nov. *(Pe.la.ga.dap.ta’tus Gr. neut. n. pelagos, the sea; adaptatus L. adjectival noun masc., adapted to; Pelagadaptatus: a microbe of the sea; a.se.a’na N. L. n. asean, derived from the acronym for Association of Southeast Asian Nations)* and SJ0813^TS^ as the sequence type for future SeqCode consideration.

While the ecological importance of *Candidatus* Pelagadaptatus aseana is unclear until an isolate is obtained, several bacterial species of *Cellvibrionaceae* such as *Sessilibacter corallicola* and *Pseudoteredinibacter isoporae* are known for their close association with corals (Chen et al., 2011; Liu et al., 2022) and other species are known for their metabolic traits of nonylphenol-degrading and xylene-utilizing (Iwaki et al., 2012; Iwaki et al., 2018). From the BLAST (blastp) searches of the RpoB, *Candidatus* Pelagadaptatus aseana is currently only detected in marine waters of the Southern Island, Singapore. Further sampling and metagenomic sequencing of coastal waters of Southeast Asia could verify its distribution in the region.

*Cellvibrionaceae* detected in MetaPhlAn was ≤0.001% (relative abundance of MetaPhlAn cluster to total MetaPhlAn cluster); while the relative abundance of our *Candidatus* Pelagadaptatus aseana MAGs to total metagenomic reads was 6.25 and 2.72%. Our finding suggests that MetaPhlAn 4, a reference-based method for taxonomic profiling grossly underestimates the abundance of *Cellvibrionaceae* taxa in the marine environment, despite incorporating MAGs of marine communities from best available sources (e.g., CAMI 2 Taxonomic profiling challenge) (Blanco-Míguez et al., 2023; Nickols et al., 2024). This highlights the need to expand the metagenome database for more environments including the marine metagenome of Southeast Asia. Hence, the approach used to estimate relative abundance of our novel MAG is to map metagenomic reads onto the MAG genome (Duru et al., 2024). The short 16S rRNA gene limited the formal proposal of our novel MAG, *Candidatus* Pelagadaptatus aseana SJ0813^TS^ under SeqCode (Hedlund et al., 2022). The 16S rRNA gene is often underrepresented in MAGs, being present in only 7 to 17.3% of them (Hiseni et al., 2022; Parks et al., 2017). Short-read assemblers do not reliably reconstruct the 16S rRNA gene owing to multiple 16S rRNA gene copies and sequence heterogeneity that hinders its correct binning with the rest of the genome (Yuan et al., 2015; Song et al., 2022). Previous studies have shown the RpoB protein as a better marker at resolving the relationships in the Gammaproteobacteria taxa than the 16S rRNA gene, hence we constructed a maximum-likelihood tree using this protein (Spring et al., 2015; Case et al., 2007).

The initial phylogeny of *U. marinipuniceus* KMM 3891^T^, an isolate from a marine mollusc, was performed using the 16S rRNA gene and subsequent proposed inclusion into *Cellvibrionaceae* was not re-evaluated using core-genome phylogenetic analysis (Spring et al., 2015; Romanenko et al., 2010). Based on the 16S rRNA gene phylogenetic tree by Romanenko et al. (2010), *U. marinipuniceus* phylogeny was unresolved with less than 90% boot-strap confidence to *Endozoicomonas elysicola* MKT 110^T^ (family *Endozoicomonadaceae*, order Oceanospirillales), *Microbulbifer* spp. (family *Microbulbiferaceae*, order Cellvibrionales) and *Simiduia agarivorans* (family *Cellvibrionaceae,* order Cellvibrionales) (Romanenko et al., 2010). This is congruent in subsequent 16S rRNA gene phylogenetic trees of the two other *Umboniibacter* species, *Umboniibacter caenipelagi* and *Umboniibacter roseus* (Sung et al., 2015; Park et al., 2017). As there is no RpoB or genome assemblies available for *U. caenipelagi* and *U. roseus,* we included the sequences of two currently undescribed *Umboniibacter* spp. strains 4–137 and HF-Din28 represented in GTDB release 220, to show that the *Umboniibacte*r spp. are indeed phylogenetically distinct from members of the *Cellvibrionaceae* and *Porticoccaceae* (Sung et al., 2015, Park et al., 2017). Physiologically, *U. marinipuniceus* is the only species known within Cellvibrionales to have Q7 as the major ubiquinone, which suggests physiological difference to the other species of Cellvibrionales (Spring et al., 2015). Unfortunately, the major respiratory lipoquinone of *P. litoralis* IMCC 2115^T^ has not been characterized (Oh et al., 2010). The congruence between the core-genome, RpoB phylogenetic trees and the monophyletic relationship of the three *Umboniibacter* species with the 16S rRNA gene supports the proposed transfer of *Umboniibacter* from the family *Cellvibrionaceae* to a newly proposed family, *Umboniibacteraceae* based on the International Code of Nomenclature of Prokaryotes (ICNP). Currently, *Umboniibacter* spp. have been isolated from benthic environments off Korea and Japan (Sung et al., 2015; Park et al., 2017; Romanenko et al., 2010). Its distribution may span to Mexico as *Umboniibacter* spp. 16S amplicons have been sequenced from oysters there (Trabal Fernández et al., 2014). The clarification of its phylogenetic placement would help future culture-dependent and -independent research in delineating the phylogeny of *Umboniibacter* species.

## 5 Description of *Umboniibacteraceae* fam. nov.

*Umboniibacteraceae* (Um.bo.ni.i.bac’ter.a.ce’ae N.L. n. *Umbonium* scientific name of a genus of marine mollusc; N.L. masc. Referring to the isolation of the first strains from the sand snail *U. costatum*; −aceae ending to denote a family; N.L. fem. pl. n. *Umboniibacteraceae* the *Umboniibacter* family).

This family is circumscribed based on core-genome phylogeny, the family belongs to the order Cellvibrionales, and the class Gammaproteobacteria. At present, the family only contains three species, *Umboniibacter marinipuniceus, Umboniibacter roseus* and *Umboniibacter caenipelagi,* all of which were isolated from marine environments such as benthic mollusks and sediments. A distinctive feature of this family is the utilization of Q7 as the major respiratory quinone. Phosphatidylethanolamine, and phosphatidylglycerol are dominant polar lipids within these species. DNA G + C content ranges from 48.9 mol% to 51.7 mol%. Major cellular fatty acid patterns are C_13:0_, C16:0 or C_17:1_ω8c. The type genus of the family is *Umboniibacter*.

## Data Availability

Sequencing raw reads and the two assembled MAGs were submitted to DNA Data Bank of Japan (DDBJ) using the DDBJ Fast Annotation and Submission Tool v. 1.6.0 (2022.3.24) under the BioProject number PRJDB17097 (Tanizawa et al., 2022). The raw reads for which the MAGs were assembled have the BioSample numbers SAMD00732050 (SJ0813; sample ID: WLagoon_S196) and SAMD00732052 (SJ0972; sample ID: WPier_S191). MAGs have the following DDBJ/GenBank accession numbers =BAABNI000000000.1 (BAABNI010000001-BAABNI010000025) for SJ0813 contigs (Biosample SAMD00738017) and =BAABNJ010000001 (BAABNJ010000001 to BAABNJ010000021) for SJ0972 contigs (Biosample SAMD00738018). The proposed nomenclature has been registered onto the SeqCode registry https://disc-genomics.uibk.ac.at/seqcode/registers/r:7vdf8rw3.

## Footnotes

^1^ https://github.com/biobakery/kneaddata

^2^ https://github.com/donovan-h-parks/CompareM

^3^ https://github.com/SilentGene/Bio-py/tree/master/POCP-calculator

^4^ https://ggdc.dsmz.de/
