# Supplementary material for: Discovery of a phylogenetically novel tropical marine Gammaproteobacteria elucidated from assembled genomes and the proposed transfer of the genus Umboniibacter from the family Cellvibrionaceae to Umboniibacteraceae fam. nov
Source: Front Microbiol. 2025 Mar 28;16:1437936. doi: 10.3389/fmicb.2025.1437936 (PMC11985809; doi:10.3389/fmicb.2025.1437936)
Supplement: Supplementary file 1 [file Supplementary_file_1.docx]

Supplementary Material

Discovery of a phylogenetically novel equatorial marine Gammaproteobacteria elucidated from metagenome-assembled genomes and the proposed transfer of the genus *Umboniibacter* from the family *Cellvibrionaceae* to *Umboniibacteraceae* fam. nov.

**Jia Yee Ho^1,2^ꝉ, Xiu Qi Koh^3^ꝉ, Deborah Yebon Kang^2^, Adrian Low^3^, Dalong Hu^1,2^, Mindia AS Haryono^2^, Rohan B. H. Williams^2^, Rebecca J. Case^4,5^, Yann Felix Boucher^1,2,6^***

^1^ Saw Swee Hock School of Public Health, National University of Singapore and National University Health System and National University Hospital System, Singapore

^2^ Singapore Centre for Environmental Life Sciences Engineering (SCELSE), National University of Singapore, Singapore

^3^ Centre for Translational Medicine, Department of Medicine, Yong Loo Lin School of Medicine, National University of Singapore, Singapore

^4^ Singapore Centre for Environmental Life Sciences Engineering (SCELSE), Nanyang Technological University, Singapore

^5^ School of Biological Sciences, Nanyang Technological University, Singapore

^6^ Infectious Diseases Translational Research Program, Department of Microbiology and Immunology, Yong Loo Lin School of Medicine, National University of Singapore and National University Hospital System, Singapore

**ꝉ These authors shared first authorship.**

*** Correspondence:**

Yann Felix Boucher (ephyb@nus.edu.sg)

# Supplementary Table

**Table S1. NCBI/DDBJ accession numbers used in the study.**

| **Strain** | **RpoB protein GenBank**  **accession no.** | **RefSeq/DDBJ**  **accession no.** | **Classification,**  **Family** |
| --- | --- | --- | --- |
| *Agaribacterium haliotis* feces2^T^ | - | GCF_002312815.1 | *Cellvibrionaceae* |
| *Agarilytica rhodophyticola* 17^T^ | - | GCF_002157225.2 | *Cellvibrionaceae* |
| *Cellvibrio mixtus* PSBB022 | - | GCF_002268635.1 | *Cellvibrionaceae* |
| *Endozoicomonas acroporae* Acr-14^T^ | WP_101748377.1 | GCF_002864045.1 | *Endozoicomonadaceae* |
| *Endozoicomonas arenosclerae* ab112^T^ | WP_062266813.1 | GCF_001562015.1 | *Endozoicomonadaceae* |
| *Endozoicomonas ascidiicola* AVMART05^T^ | WP_067516539.1 | GCF_001646945.1 | *Endozoicomonadaceae* |
| *Endozoicomonas atrinae* WP70^T^ | WP_066016062.1 | GCF_001647025.1 | *Endozoicomonadaceae* |
| *Endozoicomonas elysicola* MKT 110^T^ | WP_020581774.1 | GCF_000710775.1 | *Endozoicomonadaceae* |
| *Endozoicomonas euniceicola* EF212^T^ | WP_262595300.1 | GCF_025562755.1 | *Endozoicomonadaceae* |
| *Endozoicomonas gorgoniicola* PS125^T^ | WP_262565536.1 | GCF_025562715.2 | *Endozoicomonadaceae* |
| *Endozoicomonas montiporae* CL-33^T^ | WP_034877894.1 | GCF_001583435.1 | *Endozoicomonadaceae* |
| *Endozoicomonas numazuensis* HC50^T^ | WP_034841688.1 | GCF_000722635.1 | *Endozoicomonadaceae* |
| *Escherichia coli* ATCC 11775 | WP_000263098.1 | GCF_003697165.2 | *Enterobacteriaceae* |
| *Exilibacterium tricleocarpae* R142^T^ | - | GCF_007004655.1 | *Cellvibrionaceae* |
| *Gilvimarinus chinensis* QM 42^T^ | - | GCF_000377745.1 | *Cellvibrionaceae* |
| *Haliea salexigens* 3X/A02/235^T^ | - | GCF_000423125.1 | *Halieaceae* |
| *Halioxenophilus sp.* WMMB6 | - | GCF_033283405.1 | *Cellvibrionaceae* |
| *Marinimicrobium koreense* M9^T^ | - | GCF_003762925.1 | *Cellvibrionaceae* |
| *Microbulbifer hydrolyticus* IRE-31^T^ | WP_161859428.1 | GCF_009931115.1 | *Microbulbiferaceae* |
| *Microbulbifer agarilyticus* GP101 | WP_077406719.1 | GCF_001999945.1 | *Microbulbiferaceae* |
| *Microbulbifer salipaludis* SN0-2 | WP_207000490.1 | GCF_017303155.1 | *Microbulbiferaceae* |
| *Microbulbifer thermotolerans* JAMB A94^T^ | WP_074904364.1 | GCF_900112305.1 | *Microbulbiferaceae* |
| *Porticoccus litoralis* IMCC2115^T^ | - | GCF_030676955.1 | *Porticoccaceae* |
| *Pseudomaricurvus alkylphenolicus*KU41G^T^ | - | GCF_011683955.1 | *Cellvibrionaceae* |
| *Pseudoteredinibacter isoporae* sw-11^T^ | - | GCF_014207695.1 | *Cellvibrionaceae* |
| *Saccharophagus degradans* 2-40^T^ | - | GCF_000013665.1 | *Cellvibrionaceae* |
| *Sessilibacter corallicola* C21^T^ | - | GCF_021290925.1 | *Cellvibrionaceae* |
| *Simiduia agarivorans* SA1^T^ | - | GCF_000305785.2 | *Cellvibrionaceae* |
| SJ0813^TS^ | - | BAABNI000000000.1 | *Cellvibrionaceae* |
| SJ0972 | - | BAABNJ010000001 | *Cellvibrionaceae* |
| *Spongiibacter marinus* HAL40b^T^ | - | GCF_016906995.1 | *Spongiibacteraceae* |
| *Teredinibacter turnerae* T7902^T^ | - | GCF_000379165.1 | *Cellvibrionaceae* |
| *Umboniibacter marinipuniceus* KMM 3891^T^ | WP_121877720.1 | GCF_003688415.1 | *Umboniibacteraceae^* |
| *Umboniibacter* sp. 4-137 |  | GCA_947497555.1 | *Umboniibacteraceae^* |
| *Umboniibacter* sp. HF-Din29 |  | GCA_013372965.1 | *Umboniibacteraceae^* |

^Proposed novel family.

Figure S1. BLAST atlas comparison of our MAGs against the closest relative *Pseudomaricurvus alkylphenolicus* KU41G^T^ as reference strain where genetic similarities are shown. SJ0813 is depicted as the grey backbone, and the second ring is SJ0972. The reference strain is depicted in the third ring while the outer ring is coding sequencing (CDS) annotation with forward and reverse strands combined. Gene regions with BLAST hits are colored according to the identity scores, while the blank regions in rings 2 and 3 indicate sequencing gaps in the genome. Blank regions in the outermost ring indicate hypothetical proteins which were being removed from the annotation. rpoB gene is highlighted with red box indicating that the gene is detected in SJ0813 and reference strain, but not SJ0972.
