# Supplementary figures and images for: Discovery of a phylogenetically novel tropical marine Gammaproteobacteria elucidated from assembled genomes and the proposed transfer of the genus Umboniibacter from the family Cellvibrionaceae to Umboniibacteraceae fam. nov
Source: Front Microbiol. 2025 Mar 28;16:1437936. doi: 10.3389/fmicb.2025.1437936 (PMC11985809; doi:10.3389/fmicb.2025.1437936)

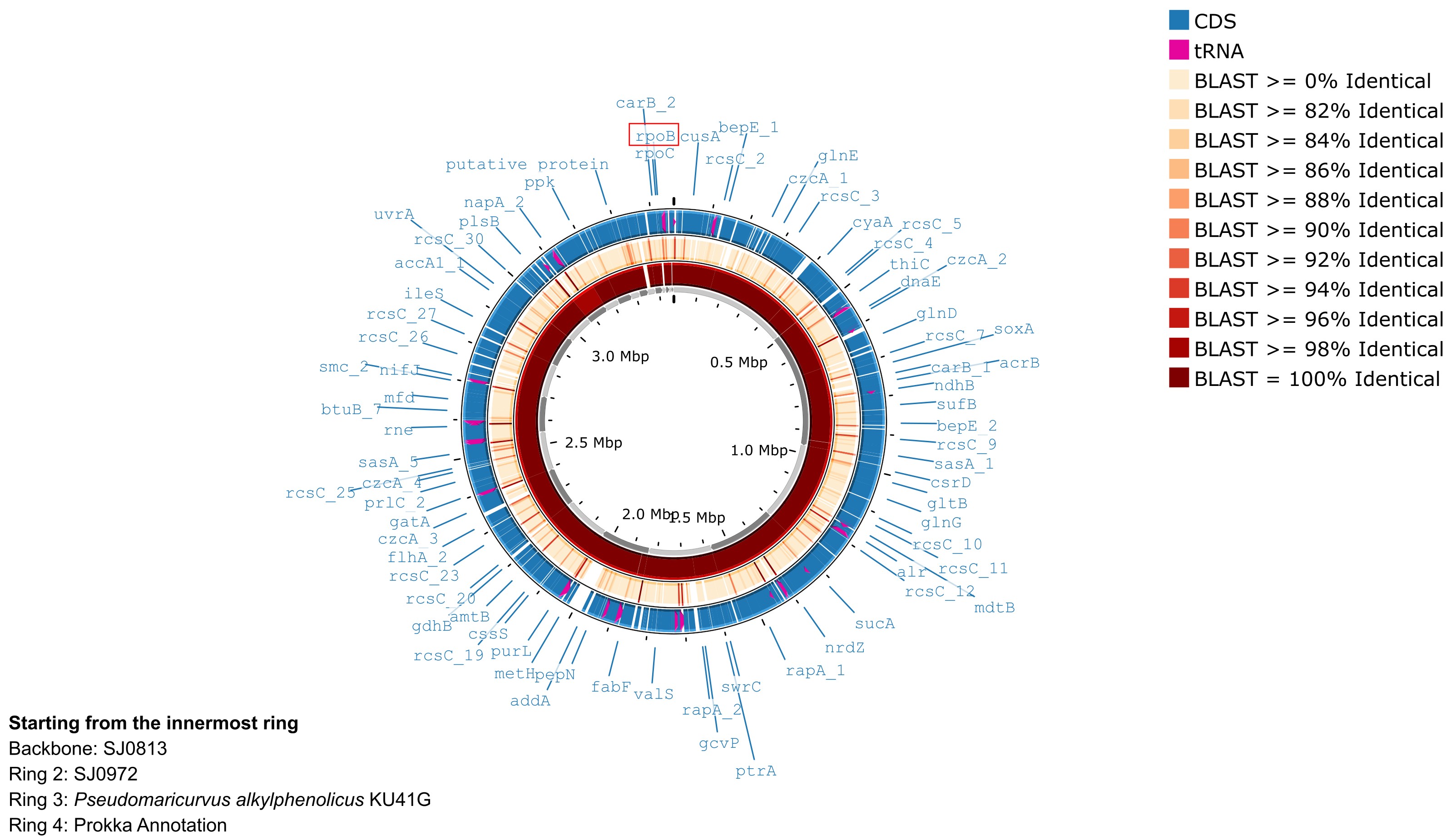

Supplement: Supplementary file 3 [file Image_1.JPEG]
